# Switching off Cancer: Is There a Role for Epigenetics?

**DOI:** 10.3390/cancers13061272

**Published:** 2021-03-12

**Authors:** Kelly A. Avery-Kiejda

**Affiliations:** 1 School of Biomedical Sciences and Pharmacy, College of Health, Medicine and Wellbeing, University of Newcastle, Callaghan, NSW 2308, Australia; Kelly.kiejda@newcastle.edu.au; 2 Hunter Medical Research Institute, New Lambton Heights, NSW 2305, Australia

Epigenetics is the study of heritable changes in gene expression that do not involve any change in DNA sequence and include methylation, histone modifications, and altered miRNA or lncRNA expression [1]. Aberrant methylation is a widespread feature in cancer that can lead to transcriptional repression of genes, which is functionally equivalent to the physical deletion of the associated gene(s). Additionally, altered expression of miRNAs and lncRNAs is common in cancer, but the consequences of this are varied and are highly dependent on the target genes regulated [2,3]. Epigenetic mechanisms of gene regulation hold a great deal of promise for cancer therapeutics, as targeting these mechanisms may provide avenues to turn oncogenes “off” or to use demethylating agents to turn tumor suppressor genes back “on”. Additionally, epigenetics holds promise for noninvasive tools to aid cancer diagnosis and inform patient prognosis because these biomarkers (methylated DNA, miRNAs, lncRNAs) are inherently stable, offering greater accessibility in formalin-fixed paraffin-embedded (FFPE) tissues and blood, ensuring minimal disruption to standard clinical practices. However, despite the rising interest and research into epigenetics in cancer, a significant clinical benefit from these studies has not been substantiated. This series of 15 articles (seven original articles, eight review articles) by international leaders in the epigenetics field explores epigenetic changes in cancer as biomarkers or potential therapies, as well as those that advance our understanding of the biological and molecular consequences of these alterations in cancer.

The methylation of DNA, the addition of a methyl group to a cytosine base in DNA, is the most widely studied epigenetic mechanism [1]. DNA methylation mostly occurs on DNA regions with at least 200 bases with at least 50% C+G content, commonly referred to as CpG islands [4]. Most human promoters are associated with CpG islands and are usually unmethylated. Cytosine bases are converted into 5-methylcytosine by DNA methyltransferase (DNMT), which generally leads to gene silencing. Several papers in this Special Issue focused on disruption of the methylome in a range of cancers including breast, lung, and prostate cancer as well as myelodysplastic disorder [5,6,7,8,9], while others have focused on the methylation of specific genes such as SFRP1 and SOX2 [10,11]. Zhang et al. comprehensively addressed the role of DNMTs in cancer in their review, while Lu and colleagues developed a novel pipeline (methylmine) for the analysis of differential methylation and demonstrated its use in TCGA methylation data [8,12].

Noncoding RNAs, including miRNAs and lncRNAs, represent a cluster of functional molecules that do not translate into proteins but play a pivotal role in the regulation of gene expression. ncRNAs are involved in numerous biological processes, and deregulation of their expression and function is observed in cancer as well as a wide range of other diseases. Yusof et al. provided an overview of ncRNAs involved in tumor-associated lymphangiogenesis and provided several possibilities for therapeutic targets [13]. Examination of lncRNAs can be challenging given their low and tissue-specific expression. In this Special Issue, Iraola-Guzman et al. developed and validated a probe-based enrichment strategy to profile lncRNAs in colorectal cancer in an effort to overcome some of these challenges [14]. More recently, RNA methylation at position N6 in adenosine (m^6^A) has been recognized as a commonly occurring epitranscriptomic modification in messenger and ncRNAs. Barros-Silva et al. showed that N6-methyladenosine modification is critical for the regulation of the oncogenic lncRNAs CCAT1 and CCAT2 in prostate cancer [15].

miRNAs are generally thought to reside within the cytoplasm where they perform their numerous cellular functions. Within this Special Issue, Bond et al. detailed how the regulation of tetraspanin by miR-518f-5p modulates breast cell migration and tumor growth, further contributing to the large body of evidence demonstrating miRNA regulation of cancer [16]. Interestingly, miRNAs have also been identified in the mitochondrion. Known as mitomiRs, they target mitochondrial or cytosolic products that modulate mitochondrion function. Ortega et al. discussed the impact of mitomiRs on breast carcinogenesis and how they could be used as therapeutic targets [17].

Together, this collection of original research articles and comprehensive reviews demonstrates the significant advancements that have already been made in the field of cancer epigenetics and opens up new avenues for unanswered questions and areas where further investigation is needed. Overall, this collection of articles underscores the importance of epigenetic regulation of cancer growth and progression and the need for further translational studies to apply this knowledge in a clinical setting either in therapies or improved methods for determining patient prognosis.
